# Authentic Leadership and Creativity: Moderated Meditation Model of Resilience and Hope in the Health Sector

**DOI:** 10.3390/ejihpe10010003

**Published:** 2019-07-17

**Authors:** Aizza Anwar, Ghulam Abid, Ali Waqas

**Affiliations:** 1 School of Professional Advancement, University of Management and Technology, Lahore 54782, Pakistan; 2 School of Business Administration, National College of Business Administration and Economics, Lahore 54660, Pakistan; 3 Department of Management Sciences, The Superior University, Lahore 55150, Pakistan

**Keywords:** authentic leadership, hope, resilience, creativity, nurses, hospitals, health sector

## Abstract

Authentic leaders are recognized as self-aware individuals who act according to their values and beliefs in the organization. Most of the studies have acknowledged the positive impact of authentic leaders on followers. However, the characteristics of an authentic leader, such as making decisions according to his/her own belief system, might negatively affect the followers. The current study aims to investigate the relationship of authenthic leadership/leaders (AL) and creativity through the mediating role of resilience. In this study, data were collected from 172 nurses working at public hospitals using a three-wave, time-lagged study design. The findings show that authentic leadership significantly predicts hope among employees. A hopeful individual positively reflects creativity in the workplace and also mediates the relationship between authentic leadership and creativity at workplace. These results report that authentic leadership impacts hope in a positive manner; thereby, increasing the level of creativity of nurses at the workplace. The study also highlights that if a resilient nurse is supervised by an authentic leader, it decreases hope and eventually creativity at work. The paper elaborates theoretical and practical implications for the health care sector along with limitations and direction for future research.

## 1. Introduction

Leadership is “a process whereby an individual influences a group of individuals to achieve a common goal” [1] and maximizes the efforts of follower, who yearn to be led by a leader [2]. There is a number of leadership styles, but no one leadership style is suitable for every situation or environment [3]. However, in the health care sector, authentic leadership has proven to be an effective approach for nurses (followers) [4]. Moreover, the recent literature on leadership is also influenced by positive psychology [5] being authentic and showing ethical behavior towards followers [6]. Authentic leadership (AL) can enhance a positive attitude among employees [7] and it is an “important organizational resource” [8]. The literature on AL [5] mentions that “authenticity has a substantial influence on how one lives one’s life”, and “ALs know who they are, what they believe and value,” and “act upon those values and beliefs while transparently interacting with others” [9]. It plays a very significant role to shape the acts of the followers at the workplace [4]; for instance, it instigates creative performance in employees [10] while building their hopes about the future [11]. It may help followers find a meaningful connection with their work [12] and assist employees to develop an affiliation with work, and do the required task in a novel way [13] because it is the responsibility of a leader to promote hope and creativity among his followers [14]. In the health care sector AL plays a key role in developing a healthy work culture for nurses [15]. Moreover, the American Association of Critical-Care Nurses (AACN) published a document that highlights AL as one of six standards critical for providing and maintaining a healthy workplace [16]. The literature on AL from scholarly perceptive highlights different philosophical approaches to improve followers’ well-being [5], where practitioner perceptive shed light on the idea that corporate crisis can be managed with the help of AL [17]. The characteristics of AL such as confidence and resilience, help leaders to act upon both the micro and macro level of an organization [18]. They are conscious about followers’ eudaemonic wellbeing [5].

AL has an ability to influence the behavior of nurses and their work outcomes [4], such as increased trust, satisfaction, and commitment [19]. It is important that the health care industry understands the value AL styles can bring to the health care sector and in improving nurses’ work effectiveness because nurses need to make important decisions related to different patients [15]. Although recent research discusses the relationship between authentic leadership and nurses, it lacks generalizability and is limited to qualitative study (interview-based) with [16] experienced nurses only across the United States [18]. It highlights a gap that future studies should investigate the relationship between AL and nurses quantitatively and extend the literature on the role of AL in healthcare [18].

Nonetheless, the majority of research work has acknowledged the positive impact of AL on employees [4,9,20,21]. However, the literature on AL [22,23] has also highlighted that AL characteristics can lead to destructive dynamics within the organization [22]. It has been argued that there may be some drawback or side effects on followers due to working with AL, for instance, being an individual who is honest and makes decision according to his/her own core belief may have negative consequences for followers [22,23]. Harvard Business Review (HBR) has highlighted that authenticity is not always a good thing because the true nature of the leader might not always in coordination with the followers [23] and the model of AL has not acknowledged the imperfections in one’s self [22].

Therefore, this research intends to fill the recent gap and extend the literature on authentic leadership, while acknowledging the fact that being an authentic leader sometimes has a negative effect on the followers. It aims to build the theoretical model that extends current views of AL as a predictor of important outcomes. It is a need of the current time that an empirical explanation of the impact of authentic leadership on nurses’ creativity and the indirect effect of hope on this relationship is provided. Moreover, the authors aim to undertake the moderating role of resilience in-between AL and hope. The article discusses recent discussion on AL; that sometimes AL cause stress for their followers [22] and in a similar manner, it is also argued that being authentic in a critical moment is also not advisable every time [24]. Thus, the aim of the current research is two-fold. Firstly, it is to empirically test the impact of AL on nurses, in the health care sector (hospitals). Secondly, it is to understand the resilience of nurses. Therefore, this study adds to the growing knowledge of authentic leadership along with its role in nurses’ creativity in the workplace.

## 2. Literature Review

### 2.1. Social Exchange Theory

Social exchange theory is a widely used theoretical framework to understand employees’ attitude and behavior in the workplace and it involves a series of interaction among individuals [25]. It elaborates the idea that “a resource will continue to flow only if there is a valued return contingent upon it” [26]. Resources are exchanged through a process of reciprocity, whereby one individual returns the good or bad actions of another individual [27]. It happens at the workplace when sometimes certain situations lead to interpersonal connections, referred to as social exchange relationships [28] The supervisor plays a very imperative role in this social exchange [29] and it has been argued by social exchange proponets that “the character of the relationship between exchange partners” might “affect the process of social exchange” [30]. Therefore, based on above discussion it is conceivable that an employee who perceives their leader’s behavior to be positive tends to be involved in more productive activity, whereas the employee who thinks otherwise will not be as productive. 

### 2.2. Individual Perception of Authentic Leadership and Hope

Leaders are trusted to be in tune with the needs of their followers, to play a protective role for followers [11], and to promote a healthy work environment [12,16]. It is also argued that positive psychological resources are inherent qualities of authentic leaders (i.e., self-knowledge) [12]. In a similar manner, characteristics of AL include the positive influence of self-awareness and self-regulated positive behaviors of leaders on followers [5] and it emphasizes on people’s strengths rather than weaknesses [8]. Similarly, authentic leaders can build trust and hope within employees [12]. However, hope is an individual’s thinking about oneself and one’s future. Hope encourages people to appraise their ability to achieve goals and overcome issues [31].

Authentic leaders are acknowledged for doing good for others through help from inner values, [32] and for positively influencing followers’ attitudes and behaviors [10]. It improves the latter’s hope, positive emotions, optimism, and trust. Additionally, such leaders provide a culture that enhances the followers’ well-being [12]. Thus, it hypothesizes that:

**H1:**  *Individual perceptions of AL are positively related to hope.*

### 2.3. Hope and Creativity

In a rapidly changing and knowledge-based economy, creativity is a necessity rather than the option. Organizations intend to change the environment and meet changing customers’ demands. AL plays a very significant role in promoting creativity among nurses in the health-care sector [4]. The creative employees assist the organizations itself by developing new ideas and suggesting novel products and procedures [33]. Authentic leadership style promotes hope and creativity within employees [34] and followers who show hope in the workplace tend to be more creative [35]. The literature on hope suggests that it leads to creativity among employees [14]. Hope is a positive emotional state that is founded as a positive approach. Agency (goal-directed energy) refers to the motivation and direction towards hopeful thinking. 

Globally, nursing experts have been encouraging nurses to pursue creativity in the workplace to improve health care outcomes. Across the world, nurses provide 80% of the primary health care [36]. Literature [7,14] suggests that positive employees who believe in themselves and their ability to manage issues are more creative. Similarly, employees who are more energetic and believe that they can meet their goals also experience more positive affect [37]. In addition, the positive attitude of employees improves their workplace creativity [38]. Thus, it hypothesizes:

**H2:**  *An employee’s hope is positively related to the employee’s creativity.*

### 2.4. Mediating Role of Hope

AL improves employees’ creativity [39]. It can be established through hope, which is identified as an intermediary element in this process [40]. Thus, it can be proposed that the positive effects of AL help inculcate hope in employees which help employees in achieving their goals while being creative at a time. A study conducted in Portuguese retail organizations reported that AL successfully predicts employees’ creativity and describes the intervening role of employees’ hope [40]. Thus, this study hypothesizes that hope can mediate the nurse’s perception of AL and their creativity:

**H3:**  *Hope mediates the relationship between individual perception of AL and creativity.*

### 2.5. Resilience as Moderator

Resilience is the capability of an individual to adapt to stressful situations or have the strength to quickly recover and deal with difficulties [41,42]. In nursing literature, the resilience construct has been explored in different studies but primarily in relation to individuals’ ability or attributes [43]. It reflects an individual’s tenacity, optimism, and aggressive approach to problem-solving and a commitment to growth from difficult situations. Individuals who are goal driven have the aility to handle adverse circumstances, and have effective problem-solving coping patterns [44]. It is important for nurses to strengthen their resilience to face difficulties and adapt to new situations along with positive future expectations [42]. Resilience was directly related to personal protective factors such as success and hope, and other emotional intelligence skills and management of feelings [45]. Therefore, the follower’s psychological capital, including resilience and self-efficacy has positive impacts on positive emotions like trust and hope of the workers [11]. 

Nurses can develop resilience by encountering stressors that they are used to facing such traumatic situations at the workplace [42] and also suggested that psychological capital (such as confidence, resilience) can influence nurses well-being in the presence of authentic [46]. The study [47] reported that resilience as an individual ability is important for nurses to moderate their reactions to the work environment and culture. AL has discussed the relationship between AL and perception of subordinate resilience, asserting that leader demonstration of moral behaviors, being more self-aware, and logical decision making improve follower resilience [48,49]. 

**Figure 1:**
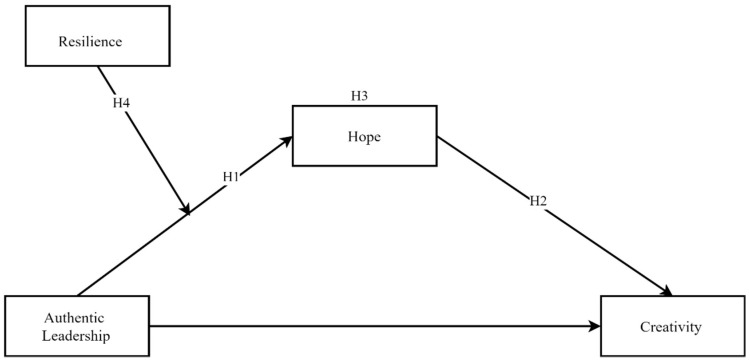
Theoretical Framework.

Although there are studies which suggest that AL has a positive relationship with the resilience of employees [50], it is not always true that having traits and characteristics of an authentic leader could become the reason for low productivity and decline in happiness [23]. It is the core characteristic of authethnic leaders to behave in harmony with their core values and beliefs [12], without fear of rejection [51]. Each individual wants to be recognized and yet to maintain his/her absolute identity [40]; as the self says, “I want to affect you, but I want nothing you do or say to affect me; I am who I am” [52]. It may happen that an AL’s core belief is not in accordance with the followers, and a hard truth shared by AL may negatively influence followers. Thus, a study [22] discussed that if leaders are encouraged to develop themselves as ALs, the results for their staff could be traumatic and could affect them psychologically. Therefore, this study hypothesized that resilience negatively moderates the relationship between AL and hope. The relationships among variables have graphically representative in the theoretical framework in Figure 1.

**H4:**  *Resilience moderates the relationship between individual perception of authentic leadership and creativity through hope.*

## 3. Materials and Methods 

### 3.1. Participants and Procedure

The respondents in this study comprised of full-time nurses working in four public government hospitals located in Lahore, Pakistan. The study emphasizes that nurses are being influenced through AL [4,19]. This study used the procedural remedy to reduce common variance bias (CMV), non-experimental three waves time-lagged survey design was used along with ensuring respondent anonymity [53]. All participants gave their informed consent for inclusion before they participated in the study. The study was approved by the committee of ethics in July of 2019. 

Data were collected in three waves, to temporally distance the predictor (AL), mediator (hope), moderator (resilience) and outcome (creativity) study variables in this study. The three surveys were administered at successive 15-day intervals. This technique ensures that there is no common method bias due to self-report data. The surveys were self-administered by one of the researchers to ensure participants could complete the surveys at the same time and place of their convenience. In the first stage of the data collection, Time 1 (T1) surveys were distributed to the nurses of the public hospitals. The nurses were provided a questionnaire after providing verbal consent to the hospital administration and were asked to fill the survey if they were willing to be part of this study. The survey instructions stressed that the survey was voluntary, and information would be used for only research purpose and kept confidential. Similarly, the same methodology was carried out by distributing questionnaires at Time 2 (T2) and Time 3 (T3). A total of 270 individuals completed the first survey, 242 individuals completed the second survey (T2), and 172 respondents completed all three surveys. Only 172 participants who completed all three surveys were included in the analyses. 

### 3.2. Measures

The study used established scales written in the English.

#### 3.2.1. Authentic Leadership

AL was measured at T1 with the 16-item scale adopted from [54]. Instructions informed respondents to think of their direct/immediate supervisor when answering the questions with four subscales: (1) self-awareness (e.g., “My supervisor Seeks feedback to improve interactions with others”), (2) relational transparency (e.g., “My supervisor says exactly what he or she means”), (3) internalized moral perspective (e.g., “Makes decisions based on his/her or her core beliefs.”), and (4) balanced processing (e.g., “My supervisor listens carefully to different points of view before coming to conclusions”). Followers rated AL on a 5-point Likert scale anchor ranging from 1 = almost never true to 5 = almost always true. The alpha of the scale was 0.92.

#### 3.2.2. Hope

Hope was assessed at T2 using [55] a 6-item scale. Instructions were given to respondents to think about their direct supervisor/leaders whom they report while answering the questions. Example items include “I can think of many ways to reach my current goals” and “There are lots of ways around any problem that I am facing right now.” Responses were collected using a 5-point Likert-type scale ranging from 1 = almost never true to 5 = almost always true. The alpha of the scale was 0.71.

#### 3.2.3. Resilience

Resilience was assessed at T2 using the [44] 4-item scale. Sample items include “I look for creative ways to alter difficult situations” and “I believe I can grow in positive ways by dealing with difficult situations.” Responses were collected using a 5-point Likert-type scale ranging from 1 = “never” to 5 = “very frequently.” The alpha of the scale was 0.78.

#### 3.2.4. Creativity

Creativity was assessed at T3 using [56] 5-item scale. Example items include “I try to be as creative as I can in my job” and “I experiment with new approaches in performing my job”. Responses were collected using a 5-point Likert-type scale ranging from 1 = not at all to 5 = extremely. It suggests how a respondent see themselves as a creative employee. The alpha of the scale was 0.88.

#### 3.2.5. Control Variables

In addition, the goal of this study was to understand the nurse’s creativity, that either they show creativity while working in an organization or not. This study included age, tenure, and marital status as a control variable.

#### 3.2.6. Tool

In this study SPSS 21 was used to analyze the data. Further, SPSS macro PROCESS [57] was used for hypothesis testing.

## 4. Results

Most of the participants were single (n = 120, 69.8%) and female (95.3%), and majority of the respondents were around 27 years old, with around 3 years of work experience. The mean, standard deviation, and an actual range for the study variables are presented in Table 1. 

The bivariate correlation between the control variables (marriage, age, tenure) and the dependent variables are presented in Table 2. In the study variables, hope was significantly correlated with AL (r = 0.20, p < 0.01) and with resilience (r = 0.50, p < 0.001). In addition, creativity showed significant correlation with resilience (r = 0.56, p < 0.001) and with hope (r = 0.23, p < 0.01). Marital status and age are not significantly correlated to any of our study variables like authentic leadership, resilience, hope, and creativity. The only significant correlations among the control variables and the outcome variables are between authentic leadership and tenure (r = 0.16, p < 0.05). The results suggests that the increase of age and change of marital status of participants has no association with their resilience, hope, and creativity. However, the increase in work experience positively and significantly influences authentic leadership.

### 4.1. Mediation Analysis

To test the mediation Model 4 was run using PROCESS [57]. Results in the “Without Moderation” row of Table 3 indicated that significant indirect effects of hope, fully mediated the association between AL and nurses’ creativity. In Step 1 of the mediation model, the regression analysis of hope with authentic leadership was significant, b = 0.16, t = 2.72, p = 0.01. Step 2 showed that the regression analysis of hope with creativity, was also significant, b = 0.37, t = 2.95, p = 0.00. Step 3 of the mediation process, regression analysis of AL and employee creativity, controlling the mediator (hope), was not significant, b = 0.04, t = 0.45, p = 0.66. According to Baron and Kenny [58], it suggests that variable hope fully mediates the relationship between AL and creativity. Additionally, a normal theory test for indirect effect found mediation in the model (z = 1.94, p = 0.05). 

### 4.2. Moderation Analysis

Results indicated in Table 3, under row entitled “With Moderation” suggest that AL and hope have a positive significant relationship (b = 1.30, t (172) = 6.52, p = 0.00). The interaction effect of AL and resilience has negative significant relationship (b = −0.33, t (172) = −6.37, p = 0.00) with hope. Moreover, the results showed that resilience and hope have a positive significant relationship (b = 1.42, t (172) = 8.37, p = 0.00). 

### 4.3. Moderated Mediation Analysis

The moderated meditation was analyzed using Model 7 of PROCESS [57] to assess the extent to which resilience moderated the meditational impact of AL on creativity through hope. 

**Figure 2:**
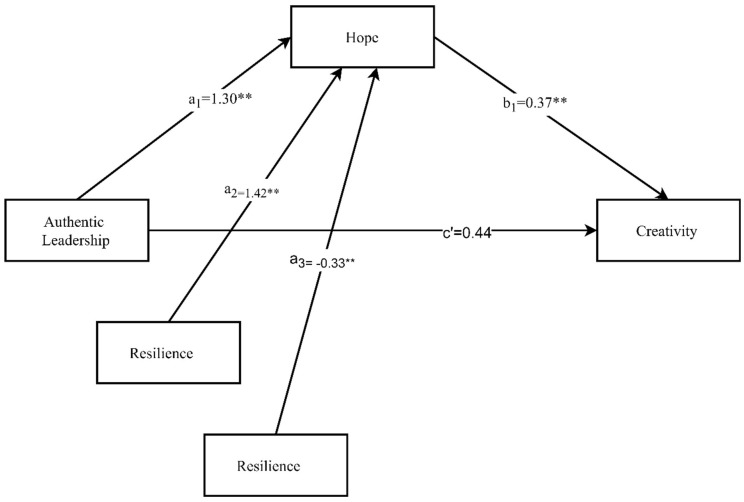
Moderated Mediation Result (Statistical Diagram).

From the moderated mediation model Figure 2 it can be seen that (1) when the association between AL and hope is modified by resilience, the mediation effect of hope is also modified; (2) this integrative model makes it possible to estimate the moderation effect of resilience on the mediator. 

In the Figure 2, coefficient values of each relationship are graphically representative using PROCESS [57] guidelines of Model 7. There is a positive significant relationship among AL and hope, resilience and hope, hope and creativity, and AL and creativity (a_1_ = 1.30 **), (a_2_ = 1.42 **), (b_1_ = 0.37 **) and (c’ = 0.44) respectively. The moderation is analyzed with the help of interaction term (authentic leadership x resilience), which has negative moderation (a_3_ = −0.33 **).

In Table 3, the confidence intervals surrounding the indirect effect of hope did not span zero, which indicates that significant indirect effect has been found at low levels of hope (β = −2.87, 95% Conf. Interval: [0.04 to 0.23], moderate levels of hope (β = 3.62, 95% Conf.Interval: [0.00 to 0.10], and high levels of hope (β = 4.38, 95% Conf. Interval: [−0.12 to −0.01). Therefore, the association between AL and creativity through hope significantly decreases when nurses have emotional resilience.

## 5. Discussion

In the proposed model, AL attributes are linked with nurses’ attitudes and behaviors in the health sector [16], and influence work outcome like creativity [4]. It was hypothesized that AL has a direct and positive relationship with hope. H1 hypothesis was found to be both positive and statistically significant. The results demonstrated strong support for H1, thus, it is accepted. It showed consistency with the previous findings [8,12]. The results of this study reflected that authentic supervisor develops hope in nurses in a way that they think in different ways to reach current goals and able to solve any problem.

The result of H2 was positive and statistically significant. Hope has a positive influence on creativity; hypothesis 2 is accepted. Previous researches supported the results of this study [7,14,35]. It implies that nurses who are more hopeful towards their ability to solve the problem are creative at work and able to overcome the barriers of the workplace. 

The H3 proposed that hope could mediate the relationship between AL and nurses’ creativity. The results of the analysis support the hypothesis. Hope variable fully mediates the AL influence on the creativity of nurses through hope; hypothesis 3 is also accepted. These findings are consistent with previous findings [40] and align with the literature [19,39]. The full mediation effect of hope in the relationship between AL and creativity indicates that nurses supervised by AL are more creative at the workplace if they opt to be hopeful while facing adversity.

The results of the interaction effect (moderation) of AL and resilience found a significant negative association with hope. This moderated mediation results implied that the nurses who are resilient, are capable to find creative ways to overcome the situation and considers the difficult situation as a mean to grow. When such nurses are supervised by authentic leaders who are known to tell the hard truth [54] and act according to his/her core belief [32] in such situation, nurses lose their hope [23]. Eventually, it has negative consequences on the creativity of nurses at the workplace. This was supported by research on the AL model which refuses to dig deep in individual imperfections [22] and its negative psychological effects on followers.

### 5.1. The Practical and Theoretical Implication

This study proposed AL impacts nurses’ creativity. These results of the study contribute theoretically to the wider body of knowledge of AL and health care sector. AL promotes hope among nurses which enable them to be a creative workforce in the hospitals. Mostly study highlights the benefits of AL on nurse’s wellbeing [14]. However, being an authentic leader can be a problem for the followers too [19] but very few studies have discussed this. The results of this study highlight that the creativity of resilient nurses decreased along with hope when they are supervised by an authentic leader. To the best of the author’s knowledge, this is the first study of its nature in a Pakistani context. 

The practical implications of the study are as follows: Firstly, AL should continue developing a ray of hope in the nurses about the future and their desire goals because it helps the nurses develop creativity. Secondly, the leader who adopts an AL style could be advised and warned against always sharing the harsh truth with their followers and, to act on their own personal core belief because it might do more damage than good to followers. This could lead to a situation where resilient nurses could eventually lose their hope and creativity. Similarly, nurses could be trained and educated on how to manage and use their resilience while working with AL, so it does not impact negatively on their workplace creativity.

### 5.2. Limitation and Future Directions

This study has a few limitations. Firstly, the participants were only from public hospitals in Lahore. It limits the interpretation of study factors in another context, such as another service sector. Therefore, future studies may use this model for other service industries to generalize findings. Secondly, the study participants were from the public sector hospitals only. Future studies can investigate the private sector or can conduct a comparison study between private and public hospitals. Thirdly, only one mediator and moderator are used in this study; future studies can use other possible mediators and moderators to explain the relationship between AL and creativity. 

Researchers are encouraged to investigate the personality traits of AL in a way to further explore, acting according to their core belief or telling the hard truth can undermine the followers work efforts and to help organizations develop AL.

## 6. Conclusions

A healthy work environment that facilitates nurses to develop creativity is a vital ingredient of quality patient care. This study proposed AL impacts on creativity via resilience and hope. In the current study, results reported that authentic leadership positively influence hope of nurses. The results implied that nurses are creative at the workplace when they are feeling hopeful about the situation. However, a relatively new perspective of AL was observed that their authenticity to act in accordance with true self and deciding according to their core belief has negative consequences for resilient nurses. HBR has also questioned AL characteristics of being real and its negative influence on followers. In a similar manner, the workplace creativity of resilient nurses decreases when they are supervised by a leader who acts upon their true self. These results are consistent with social exchange theory that employee’s perception about their leader’s behavior tends to reflect in their workplace outcome of employees.

## Figures and Tables

**Table 1:** Descriptive Statistics for the Study Variables (N = 172).

Variables	Mean	SD	Range
1. Marital Status	1.30	0.46	1–2
2. Age	27.33	4.07	21–45
3. Tenure	3.56	3.15	1–17
4. Authentic Leadership	3.35	0.74	1.38–4.94
5. Resilience	3.62	0.76	1.25–5.0
6. Hope	3.89	0.59	1.17–4.83
7. Creativity	3.81	0.96	1.20–5.0

**Table 2:** The Correlation Matrix for Study Variables.

Variables	Marital Status	Age	Tenure	Authentic Leadership	Resilience	Hope	Creativity
Marital Status	1						
Age	0.48 **	1					
Tenure	0.38 **	0.72 **	1				
Authentic Leadership	−0.01	0.09	0.16 *	(0.92)			
Resilience	0.05	−0.03	−0.01	0.10	(0.71)		
Hope	0.02	−0.09	−0.03	0.20 **	0.50 **	(0.78)	
Creativity	0.06	0.08	0.01	0.08	0.56 **	0.23 **	(0.88)

* p < 0.05 ** p< 0.01 Cronbach’s alpha values given in parentheses.

**Table 3:** Unstandardized Regression Coefficients with Confidence Intervals.

Analysis	Hope (M)		Creativity (Y)	
	Coeff.	95% CI	Coeff.	95% CI
**Without Moderation**				
Authentic Leadership (X)	0.16	0.04, 0.28	0.04	−0.15, 0.24
Hope (M)			0.37	0.12, 0.61
**With Moderation**				
Authentic Leadership (X)	1.30	0.93, 1.68	0.04	−0.15, 0.24
Hope (M)			0.37	0.12, 0.61
Resilience(W)	1.42	1.09, 1.76		
X x W	−0.33	−0.43, −0.23		
R2	0.41		0.06	
F	40.32		4.93	

Note: Confidence interval with no zero in the range is significant.
